# A second monoclinic polymorph of ferrocenecarboxaldehyde

**DOI:** 10.1107/S205698902600229X

**Published:** 2026-03-05

**Authors:** Jamal Lasri, Yaseen A. Almehmadi, Naser E. Eltayeb, Tuncer Hökelek, Aidan P. McKay

**Affiliations:** a Department of Chemistry Rabigh College of Science and Arts King Abdulaziz University,Jeddah 21589 Saudi Arabia; b King Fahd Medical Research Center King Abdulaziz University,Jeddah 21589 Saudi Arabia; c Department of Chemistry, Faculty of Pure and Applied Sciences, International University of Africa, Khartoum 2469, Sudan; d Department of Physics, Hacettepe University, 06800 Beytepe, Ankara, Türkiye; e EaStCHEM School of Chemistry, University of St Andrews, Fife KY16 9ST, United Kingdom; Universidade Federal do ABC, Brazil

**Keywords:** ferrocenecarboxaldehyde, crystal structure, hydrogen bond, π-stacking, Hirshfeld surface

## Abstract

The title compound, [Fe(C_5_H_5_)(C_6_H_5_O)] (**I**), crystallizes in the space group *P*2_1_ with two crystallographically independent ferrocenecarboxaldehyde mol­ecules in the asymmetric unit, in which the C—O bond lengths and also the O—C—C bond angles of the carboxaldehyde moieties have significantly different values. In the crystal, C—H⋯O hydrogen bonds link the mol­ecules into infinite chains along the *b*-axis direction. The π–π stacking inter­actions between the parallel ferrocene rings [centroid-to-centroid distances of 3.305 (4) and 3.293 (4) Å] and the C—H⋯π(ring) inter­actions help to consolidate the packing. Compound **I** is a polymorph of the previously reported form of ferrocenecarboxaldehyde [Sato *et al.* (1984 ▸). *Bull. Chem. Soc. Jpn***57**, 634–638; Lousada *et al.* (2008 ▸). *J. Phys. Chem. A.***112**, 2977–2987], which crystallizes in the space group *P*2_1_2_1_2_1_ with one mol­ecule in the asymmetric unit. The Hirshfeld surface analysis of the crystal structure indicates that the most important contributions for the crystal packing are from H⋯H (54.8%), H⋯C/C⋯H (26.5%) and H⋯O/O⋯H (18.4%) inter­actions. The volume of the crystal voids and the percentage of free space were calculated to be 53.38 Å^3^ and 6.03%, showing that there is no large cavity in the crystal packing. Hydrogen bonding, π–π, C—H⋯π(ring) and van der Waals inter­actions are the dominant inter­actions in the crystal packing.

## Abstract 2

The asymmetric unit of the title compound contains two crystallographically independent ferrocenecarboxaldehyde mol­ecules. In the crystal, C—H⋯O hydrogen bonds link the mol­ecules into infinite chains along the *b*-axis direction. The hydrogen bonding, π–π, C—H⋯π(ring) and van der Waals inter­actions are the dominant inter­actions in the crystal packing.

## 1. Chemical context

Since its discovery in 1951, compounds containing the ferrocene moiety have been of significant inter­est due to their application in environmental pollution remediation (Wang *et al.*, 2014 ▸; Kaur *et al.*, 2015 ▸). The well-established chemistry of ferrocene derivatives along with their stabilities encouraged their incorporation in the synthesis of materials with non-linear optical (Di Bella *et al.*, 2001 ▸) or reversible redox properties (Kowalski *et al.*, 2014 ▸) or they can be used as catalysts (Ruble *et al.*, 1997 ▸). Moreover, biologically active materials containing ferrocene as a modified Tamoxifen drug by replacing one π–π group of the aromatic rings with a ferrocene fragment have been developed (Top *et al.*, 2001 ▸). Reasonable anti­malarial activity was noted against *Plasmodium falciparum*, even against those that are chloro­quine resistant (Biot *et al.*, 2006 ▸). Ferrocene-based asymmetrical azines have shown potential as anti­microbial-anti­tumor agents (Lasri *et al.*, 2018*a* ▸). Moreover, ferrocene-based Schiff bases have been found to be good absorbents for methyl blue from water (Lasri *et al.*, 2018*b* ▸). Herein we report the mol­ecular and crystal structures, Hirshfeld surface analysis and crystal voids of the title compound. Compound **I** is a polymorph of the previously reported form of ferrocenecarboxaldehyde [Sato *et al.*, 1984 ▸; Lousada *et al.*, 2008 ▸; Cambridge Structural Database (CSD; Groom *et al.*, 2016 ▸) refcodes DEJZAT and DEJZAT01, respectively] in the space group *P*2_1_2_1_2_1_ with one mol­ecule in the asymmetric unit.
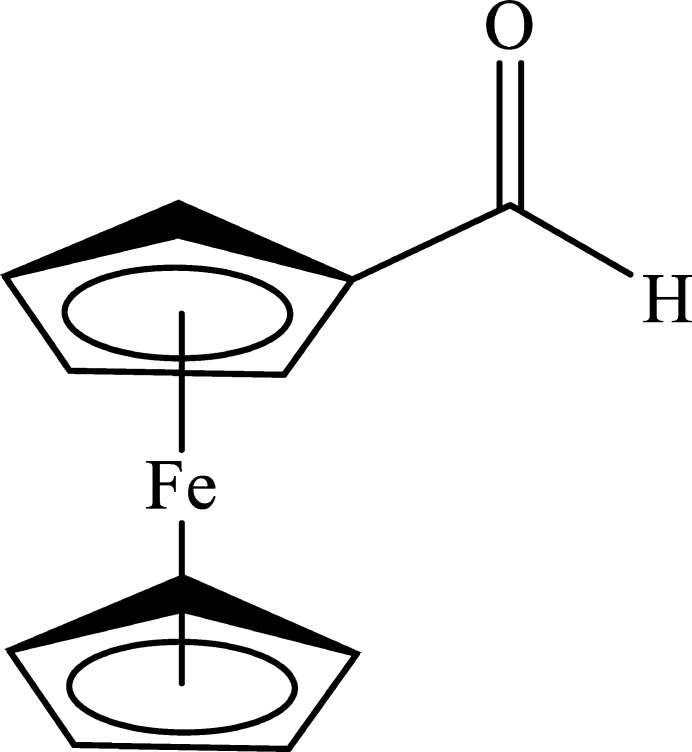


## 2. Structural commentary

**Figure 1:**
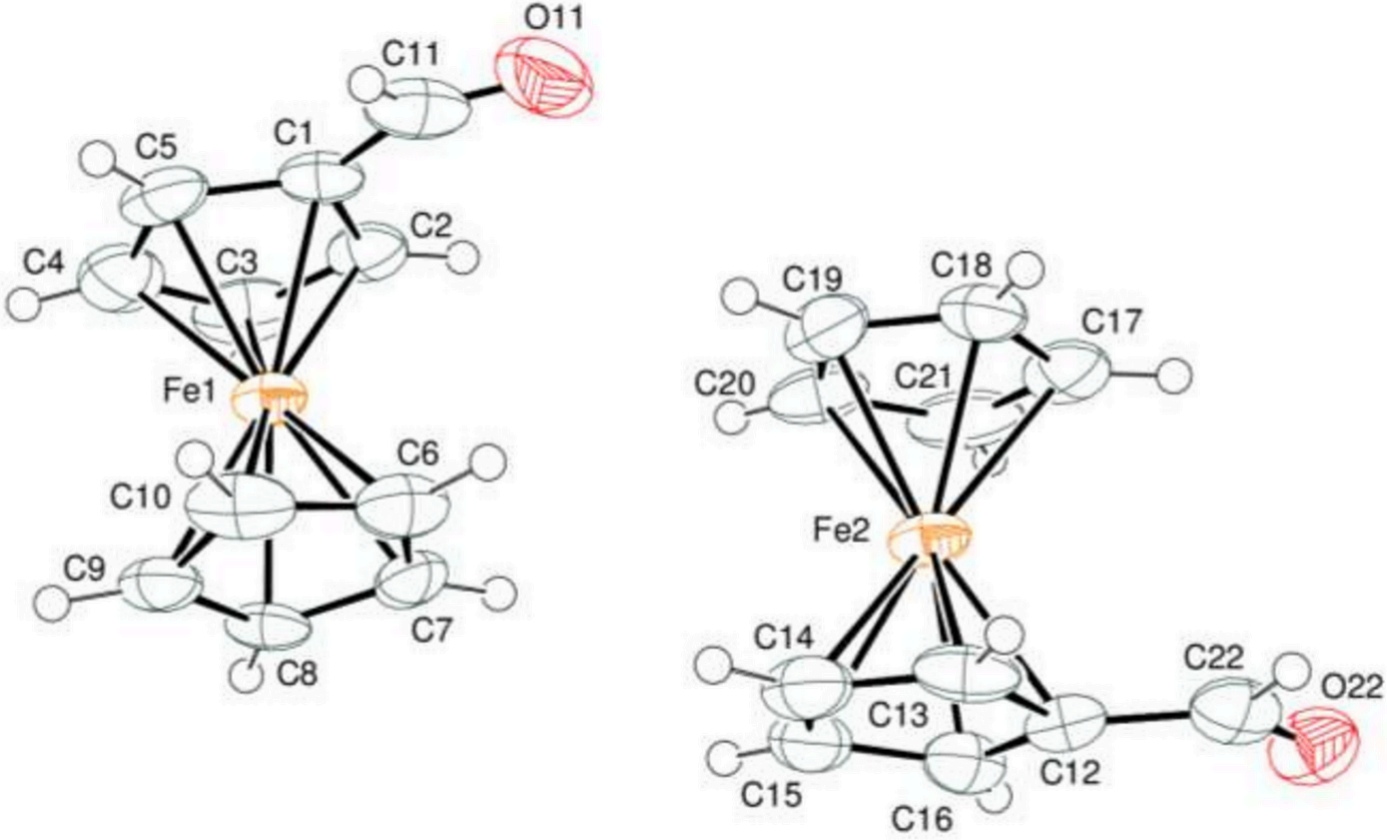
The title mol­ecule with the atom-numbering scheme and 50% probability ellipsoids.

The asymmetric unit of the title compound contains two crystallographically independent ferrocenecarboxaldehyde mol­ecules (Fig. 1 ▸). In the carboxaldehyde moieties, the C11—O11 [1.137 (14) Å] and C22—O22 [1.229 (12) Å] bond lengths and also the O11—C11—C1 [130.2 (13)°] and O22—C22—C12 [125.6 (9)°] bond angles have significantly different values. The corresponding values are C11—O11 = 1.042 (10) Å and O11—C11—C6 = 142.5 (17)° in the previously reported form of ferrocenecarboxaldehyde (Lousada *et al.*, 2008 ▸). On the other hand, the C1—C11 [1.451 (16) Å] and C12—C22 [1.442 (14) Å] bond lengths are between the typical values of single and double C—C bonds of 1.54 and 1.40 Å, respectively, supporting the existence of CO—Cp conjugation. The corresponding C—C bond was reported as C6—C11 = 1.444 (14) Å in the previously reported form of ferrocenecarboxaldehyde (Lousada *et al.*, 2008 ▸).

The C5—C1—C11—O11 [−173.5 (10)°], C2—C1—C11—O11 [15.6 (18)°] and C13—C12—C22—O22 [−177.4 (9)°], C16—C12—C22—O22 [−6.0 (16)°] torsion angles indicate that the CHO substituents are almost coplanar with the Cp rings, thus allowing conjugations of the π–π electron systems of the C=O bonds and the aromatic cyclo­penta­dienyl rings. Atoms O11, C11 and O22, C22 are 0.065 (6), 0.152 (7) and 0.167 (4), 0.128 (6) Å, respectively, away from the corresponding best least-squares ring planes. Thus, they are almost coplanar with the adjacent Cp rings. The planar *A* (C1–C5), *B* (C6–C10) and *C* (C12—16), *D* (C17–C21) rings are oriented at dihedral angles of *A*/*B* = 1.15 (8)°, *A*/*C* = 13.98 (27)°, *A*/*D* = 13.55 (27)°, *B*/*C* = 14.30 (25)°, *B*/*D* = 13.84 (28)° and *C*/*D* = 0.64 (22)°.

The Fe1—C and Fe2—C bond lengths are within the ranges 2.016 (9)—2.056 (10) Å and 2.021 (10)—2.056 (11) Å, respectively, for the two independent mol­ecules in the asymmetric unit. The C1—C11 [1.451 (16) Å] and C12—C22 [1.442 (14) Å] bond lengths are similar but the O11—C11 [1.137 (14) Å] bond is shorter than the C22—O22 [1.229 (12) Å] bond. On the other hand, the C12—C22—O22 [125.6 (9)°] bond angle is narrower than the corresponding C1—C11—O11 [130.2 (13)°] bond angle.

## 3. Supra­molecular features

**Figure 2:**
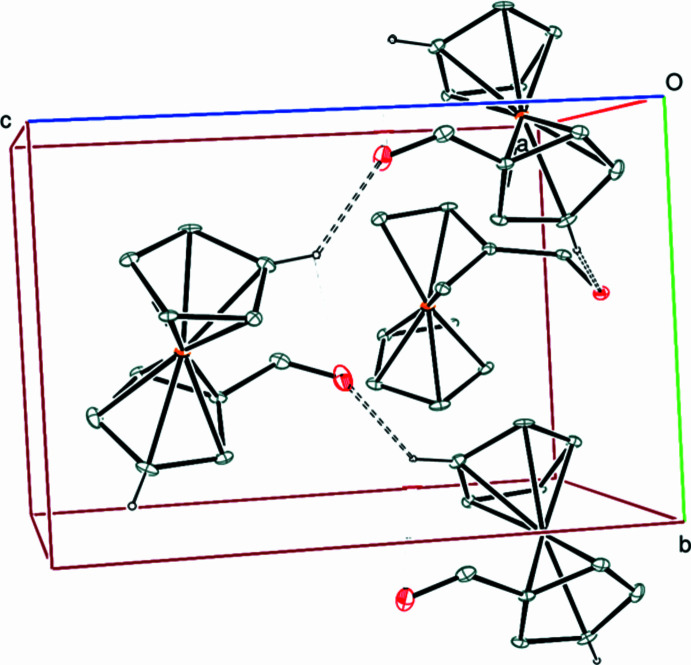
A partial packing diagram viewed down the *a*-axis direction. C—H⋯O hydrogen bonds are shown as dashed lines. The (C)—H atoms not involved in hydrogen bonds have been omitted for clarity.

In the crystal, C6—H6⋯O11 hydrogen bonds (Table 1 ▸) link the mol­ecules into infinite chains along the *b*-axis direction (Fig. 2 ▸), and C3—H3⋯O22 hydrogen bonds (Table 1 ▸) link the mol­ecules to these chains (Fig. 2 ▸). There are π–π stacking inter­actions between the parallel ferrocene rings with centroid-to-centroid distances of 3.305 (7) and 3.293 (7) Å. The C—H⋯π(ring) inter­actions (Table 2 ▸) may help to consolidate the packing. Hydrogen bonding, C—H⋯π(ring) and van der Waals inter­actions are the dominant inter­actions in the crystal packing.

## 4. Hirshfeld surface analysis

**Figure 3:**
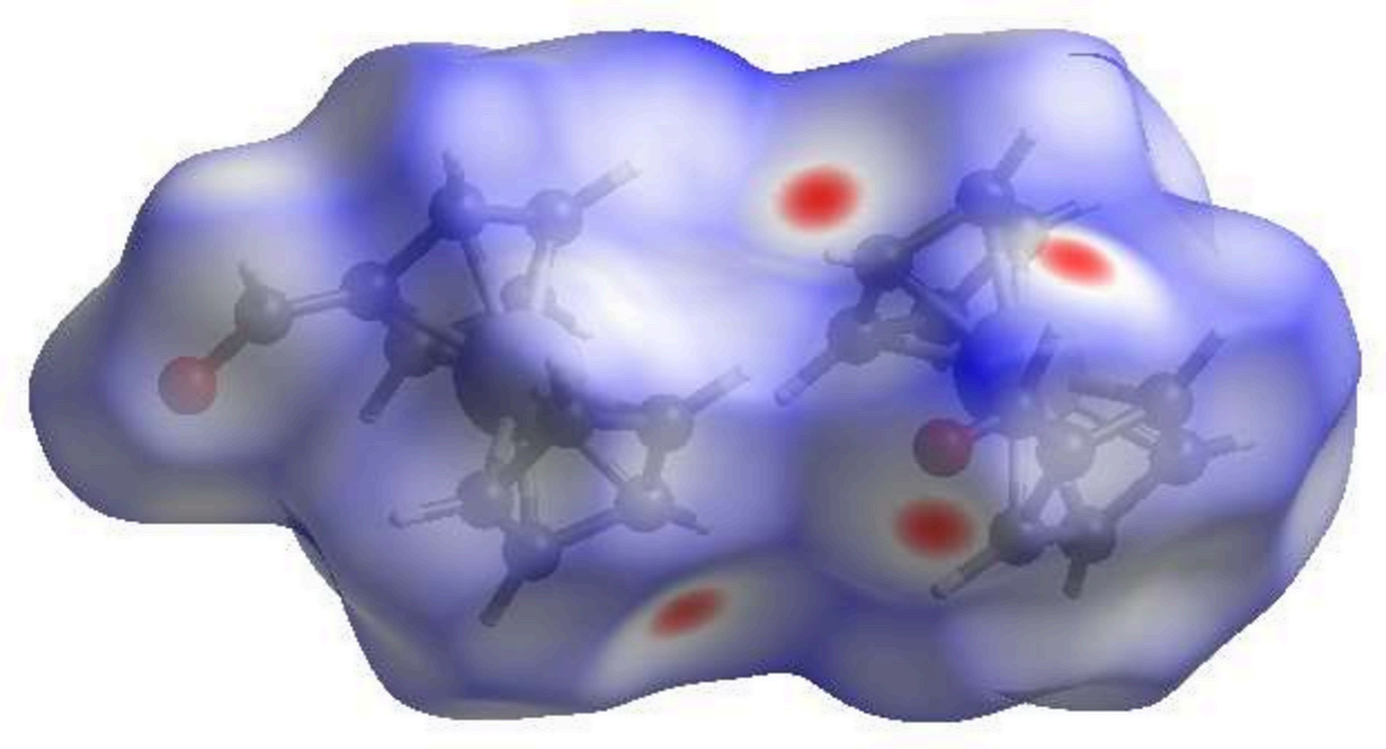
View of the three-dimensional Hirshfeld surface of the title compound plotted over *d*_norm_.

**Figure 4:**
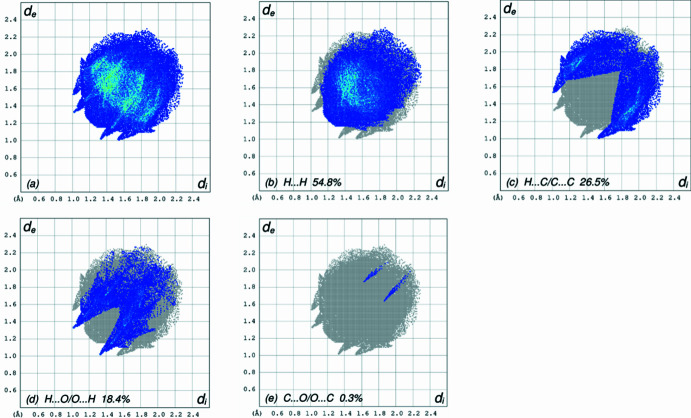
The full two-dimensional fingerprint plots for the title compound, showing (*a*) all inter­actions, and delineated into (*b*) H⋯H, (*c*) H⋯C/C⋯H, (*d*) H⋯O/O⋯H and (*e*) C⋯O/O⋯C, inter­actions. The *d*_i_ and *d*_e_ values are the closest inter­nal and external distances (in Å) from given points on the Hirshfeld surface.

A Hirshfeld surface (HS) analysis was carried out by *Crystal Explorer 17.5* (Spackman *et al.*, 2021 ▸) to clarify the inter­molecular inter­actions in the crystal. The contact distances (Table 1 ▸) are shown in Fig. 3 ▸, where the bright-red spots correspond to the respective donors and/or acceptors. According to the 2D fingerprint plots (McKinnon *et al.*, 2007 ▸), the inter­molecular H⋯H, H⋯C/C⋯H and H⋯O/O⋯H contacts make important contributions to the HS of 54.8%, 26.5% and 18.4%, respectively (Fig. 4 ▸).

## 5. Database survey

A survey of the Cambridge Structural Database (CSD, July 2025 update; Groom *et al.*, 2016 ▸) revealed seven structures containing the target compound ferrocenecarboxaldehyde **I** (DEJZAT; Sato *et al.*, 1984 ▸), compound **II** (DEJZAT01; Lousada *et al.*, 2008 ▸), compound **III** (GUCJIA; Singh *et al.*, 2020 ▸), compound **IV** (MEJMUK Kim *et al.*, 2006 ▸) compound **V** (QARLON Brunet *et al.*, 2017 ▸), compound **VI** (QONQEQ Meilikhov *et al.*, 2009 ▸), compound **VII** (XUFCEJ Zhang *et al.*, 2020 ▸).

## 6. Crystal voids

**Figure 5:**
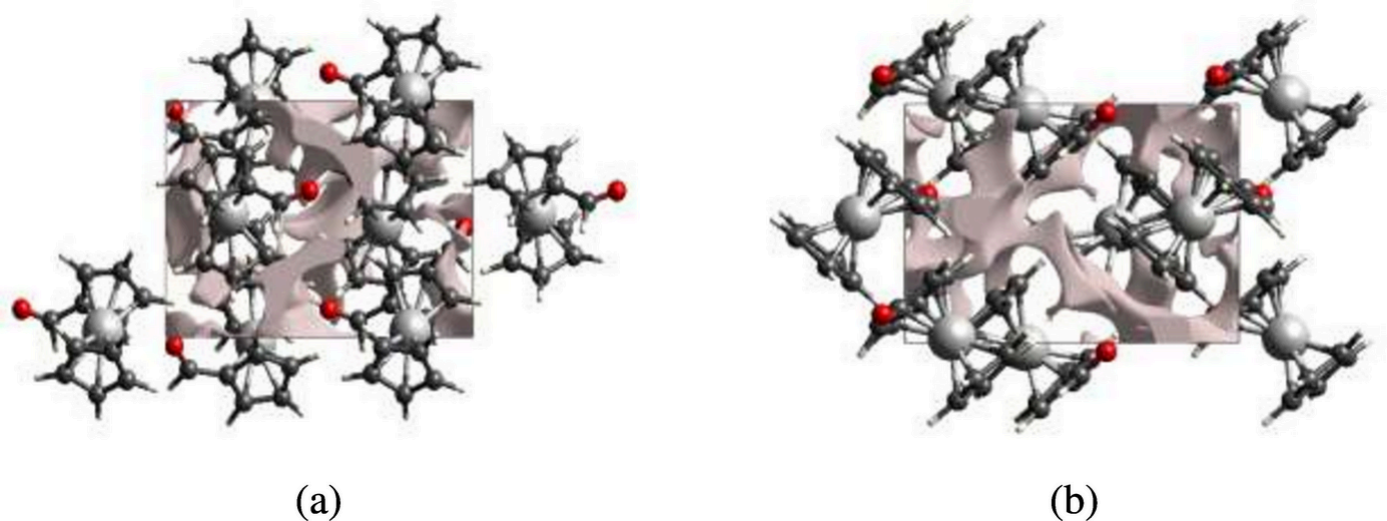
Graphical views of the voids in the crystal packing of the title compound. (*a*) along *a*-axis and (*b*) along *c*-axis directions.

If the mol­ecules are tightly packed and the applied external mechanical force does not easily break the crystal, then the crystal packing does not result in significant voids. A void analysis was performed by adding up the electron densities of the spherically symmetric atoms contained in the asymmetric unit (Turner *et al.*, 2011 ▸). The volume of the crystal voids (Fig. 5 ▸*a* and *b*) and the percentage of free space in the unit cell are calculated as 53.38 Å^3^ and 6.03%, respectively, indicating that the crystal packing is compact.

## 7. Synthesis and crystallization

To a solution of *N*-methyl­hydroxyl­amine hydro­chloride (100.0 mg, 1.20 mmol) in MeOH (50 ml) was added sodium carbonate (63.4 mg, 0.60 mmol) and the reaction mixture was stirred for 10 min followed by the addition of ferrocenecarboxaldehyde (232.9 mg, 1.09 mmol). Then, the mixture was stirred for 12 h at room temperature. After that, the precipitate formed was filtered off. The expected product *N*-methyl-*C*-ferrocenyl aldo­nitrone was not detected, in contrast, only the starting material ferrocenecarboxaldehyde was recuperated. Orange crystals suitable for X-ray analysis were obtained by slow evaporation of a methanol solution at room temperature.

**Fe[(η^5^-C_5_H_5_)(η^5^-C_5_H_4_CHO)]**. FT-IR (cm^−1^) 3086 (ν_C–H_, C_5_H_5_); 2865, 2832, 2803, 2761 and 2726 (ν_C–H_, CHO); 1681 (ν_C–O_, CHO); 1104 and 1409 (ν_C–C_, C_5_H_5_); 1387 (δ_C–H_, CHO); 1002 (δ_C–H_, C_5_H_5_); 824 and 842 (π_C–H_, C_5_H_5_); 497 (C_5_H_5_ ring tilt); 481 (ν_Fe–C_5_H_5__). ^1^H NMR (CDCl_3_): δ 4.28 (5H, C_5_H_5_); 4.61 (2H, C_5_H_4_) and 4.80 (2H, C_5_H_4_); 9.96 (1H, CH=O). Elemental analysis for C_11_H_10_OFe: calculated, C 61.73%, H 4.71%; found, C 61.50%, H 4.57%. The observed FT-IR and ^1^H NMR spectra are in good agreement with those reported (Lousada *et al.*, 2008 ▸).

## 8. Refinement

Crystal data, data collection and structure refinement details are summarized in Table 1 ▸.. The C-bound hydrogen atom positions were calculated geometrically at distances of 0.95 Å (for aromatic and methine CH) and refined using a riding model by applying the constraint *U*_iso_(H) = 1.2 × *U*_eq_(C). Data were processed as a two-component twin with the second component rotated by 179.9856° around [0 0 1] (reciprocal), or [0.05 0 1] (direct) and the twin component ratio was refined to 0.501:0.499. The Flack absolute structure parameter (Parsons *et al.*, 2013 ▸) refined to −0.03 (2). The expected values are 0.00 and 1.00 for correct and reverse absolute structures, respectively. Thus, the absolute structure was determined unambiguously.

## Supplementary Material

Crystal structure: contains datablock(s) I. DOI: 10.1107/S205698902600229X/ee2027sup1.cif

Structure factors: contains datablock(s) I. DOI: 10.1107/S205698902600229X/ee2027Isup2.hkl

CCDC reference: 2534450

Additional supporting information:  crystallographic information; 3D view; checkCIF report

## Figures and Tables

**Table 1:** Hydrogen-bond geometry (Å, °) *Cg*1–*Cg*4 are the centroids of the (C1–C5), (C6–C10), (C12–C16) and (C17–C21) rings, respectively.

*D*—H⋯*A*	*D*—H	H⋯*A*	*D*⋯*A*	*D*—H⋯*A*
C3—H3⋯O22^i^	0.95	2.60	3.520 (13)	165
C6—H6⋯O11^ii^	0.95	2.45	3.255 (13)	142
C7—H7⋯*Cg*3^i^	0.95	2.91	3.625 (12)	133
C11—H11⋯*Cg*4^ii^	0.95	2.97	3.907 (12)	170
C16—H16⋯*Cg*2^iii^	0.95	3.03	3.935 (13)	159
C18—H18⋯*Cg*1^ii^	0.95	2.80	3.631 (7)	146

Symmetry codes: (i) 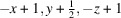; (ii) 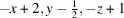; (iii) 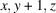.

**Table 2:** Experimental details

Crystal data	
Chemical formula	[Fe(C_5_H_5_)(C_6_H_5_O)]
*M*_r_	214.04
Crystal system, space group	Monoclinic, *P*2_1_
Temperature (K)	173
*a*, *b*, *c* (Å)	10.4438 (9), 7.5766 (8), 11.2044 (14)
β (°)	92.459 (15)
*V* (Å^3^)	885.77 (17)
*Z*	4
Radiation type	Mo *K*α
μ (mm^−1^)	1.65
Crystal size (mm)	0.1 × 0.09 × 0.02

Data collection	
Diffractometer	Rigaku XtaLAB P200K
Absorption correction	Multi-scan (*CrysAlis PRO*; Rigaku OD, 2024 ▸)
*T*_min_, *T*_max_	0.725, 1.000
No. of measured, independent and observed [*I* > 2σ(*I*)] reflections	6451, 6451, 5568
(sin θ/λ)_max_ (Å^−1^)	0.699

Refinement	
*R*[*F*^2^ > 2σ(*F*^2^)], *wR*(*F*^2^), *S*	0.054, 0.145, 1.07
No. of reflections	6451
No. of parameters	236
No. of restraints	1
H-atom treatment	H-atom parameters constrained
Δρ_max_, Δρ_min_ (e Å^−3^)	1.14, −0.49
Absolute structure	Flack *x* determined using 1494 quotients [(*I*^+^)−(*I*^−^)]/[(*I*^+^)+(*I*^−^)] (Parsons et al., 2013 ▸)
Absolute structure parameter	−0.03 (2)

Computer programs: *CrysAlis PRO* (Rigaku OD, 2024 ▸), *SHELXT* (Sheldrick, 2015*a* ▸), *SHELXL2019/3* (Sheldrick, 2015*b* ▸) and *OLEX2* (Dolomanov *et al.*, 2009 ▸).

## supplementary crystallographic information

### Ferrocenecarboxaldehyde Crystal data

**Table:**

[Fe(C_5_H_5_)(C_6_H_5_O)]	*F*(000) = 440
*M**_r_* = 214.04	*D*_x_ = 1.605 Mg m^−^^3^
Monoclinic, *P*2_1_	Mo *K*α radiation, λ = 0.71073 Å
*a* = 10.4438 (9) Å	Cell parameters from 4087 reflections
*b* = 7.5766 (8) Å	θ = 2.6–26.3°
*c* = 11.2044 (14) Å	µ = 1.65 mm^−^^1^
β = 92.459 (15)°	*T* = 173 K
*V* = 885.77 (17) Å^3^	Plate, orange
*Z* = 4	0.1 × 0.09 × 0.02 mm

### Ferrocenecarboxaldehyde Data collection

**Table:**

Rigaku XtaLAB P200K diffractometer	6451 measured reflections
Radiation source: Rotating Anode, Rigaku FR-X	6451 independent reflections
Rigaku Osmic Confocal Optical System monochromator	5568 reflections with *I* > 2σ(*I*)
Detector resolution: 5.8140 pixels mm^-1^	θ_max_ = 29.8°, θ_min_ = 1.8°
shutterless scans	*h* = −14→14
Absorption correction: multi-scan (CrysAlisPro; Rigaku OD, 2024)	*k* = −10→10
*T*_min_ = 0.725, *T*_max_ = 1.000	*l* = −15→14

### Ferrocenecarboxaldehyde Refinement

**Table:**

Refinement on *F*^2^	Hydrogen site location: inferred from neighbouring sites
Least-squares matrix: full	H-atom parameters constrained
*R*[*F*^2^ > 2σ(*F*^2^)] = 0.054	*w* = 1/[σ^2^(*F*_o_^2^) + (0.0947*P*)^2^ + 0.3203*P*] where *P* = (*F*_o_^2^ + 2*F*_c_^2^)/3
*wR*(*F*^2^) = 0.145	(Δ/σ)_max_ < 0.001
*S* = 1.07	Δρ_max_ = 1.14 e Å^−^^3^
6451 reflections	Δρ_min_ = −0.49 e Å^−^^3^
236 parameters	Absolute structure: Flack *x* determined using 1494 quotients [(*I*^+^)-(*I*^-^)]/[(*I*^+^)+(*I*^-^)] (Parsons et al., 2013)
1 restraint	Absolute structure parameter: −0.03 (2)
Primary atom site location: dual	

### Ferrocenecarboxaldehyde Special details

**Table:**

Geometry. All esds (except the esd in the dihedral angle between two l.s. planes) are estimated using the full covariance matrix. The cell esds are taken into account individually in the estimation of esds in distances, angles and torsion angles; correlations between esds in cell parameters are only used when they are defined by crystal symmetry. An approximate (isotropic) treatment of cell esds is used for estimating esds involving l.s. planes.
Refinement. Data was processed as 2-component twin with second component rotated 179.9856 ° around [0 0 1] (reciprocal), or [0.05 0 1] (direct) and twin component ratio was refined to 0.501:0.499

### Ferrocenecarboxaldehyde Fractional atomic coordinates and isotropic or equivalent isotropic displacement parameters (Å^2^)

**Table:**

	*x*	*y*	*z*	*U*_iso_*/*U*_eq_	
Fe1	0.85974 (10)	0.52362 (12)	0.79568 (10)	0.0334 (3)	
Fe2	0.63769 (10)	0.47927 (12)	0.29292 (10)	0.0355 (3)	
O11	1.0675 (8)	0.6185 (16)	0.5310 (8)	0.090 (4)	
O22	0.3966 (6)	0.4636 (10)	0.0285 (6)	0.0536 (16)	
C1	1.0167 (8)	0.6417 (14)	0.7329 (9)	0.041 (2)	
C2	0.9145 (8)	0.7685 (14)	0.7382 (10)	0.046 (2)	
H2	0.877080	0.833524	0.673149	0.055*	
C3	0.8811 (10)	0.7776 (15)	0.8563 (12)	0.059 (3)	
H3	0.814826	0.849431	0.885565	0.071*	
C4	0.9594 (11)	0.666 (2)	0.9255 (10)	0.065 (3)	
H4	0.957367	0.650632	1.009600	0.078*	
C5	1.0422 (8)	0.5783 (15)	0.8491 (10)	0.050 (3)	
H5	1.104310	0.491776	0.872161	0.060*	
C6	0.7910 (9)	0.3445 (16)	0.6723 (10)	0.049 (3)	
H6	0.819576	0.328703	0.593687	0.058*	
C7	0.6925 (8)	0.4557 (16)	0.7051 (8)	0.042 (2)	
H7	0.643260	0.529986	0.652538	0.051*	
C8	0.6781 (7)	0.4405 (11)	0.8278 (8)	0.038 (2)	
H8	0.617244	0.501784	0.872952	0.046*	
C9	0.7696 (8)	0.3181 (14)	0.8732 (10)	0.044 (2)	
H9	0.780953	0.282107	0.954190	0.053*	
C10	0.8414 (9)	0.2583 (14)	0.7771 (11)	0.051 (3)	
H10	0.910234	0.176307	0.781461	0.061*	
C11	1.0735 (9)	0.5704 (17)	0.6272 (11)	0.059 (3)	
H11	1.123685	0.467074	0.640538	0.071*	
C12	0.4993 (8)	0.3378 (15)	0.2019 (8)	0.038 (2)	
C13	0.5964 (9)	0.2244 (13)	0.2545 (10)	0.046 (2)	
H13	0.649654	0.147055	0.211912	0.055*	
C14	0.6000 (9)	0.2460 (16)	0.3784 (10)	0.052 (3)	
H14	0.654411	0.184722	0.434459	0.062*	
C15	0.5080 (9)	0.3757 (15)	0.4053 (9)	0.049 (2)	
H15	0.491131	0.417869	0.482939	0.059*	
C16	0.4453 (9)	0.4321 (15)	0.2973 (8)	0.045 (2)	
H16	0.379047	0.517771	0.289947	0.054*	
C17	0.7123 (10)	0.6572 (16)	0.1795 (11)	0.053 (3)	
H17	0.686053	0.673903	0.098046	0.064*	
C18	0.8094 (7)	0.5402 (17)	0.2229 (8)	0.046 (2)	
H18	0.859414	0.463800	0.176419	0.055*	
C19	0.8176 (8)	0.5585 (16)	0.3472 (9)	0.051 (3)	
H19	0.875779	0.496898	0.399699	0.061*	
C20	0.7268 (9)	0.6817 (16)	0.3823 (12)	0.057 (3)	
H20	0.711420	0.717072	0.461760	0.068*	
C21	0.6620 (10)	0.7436 (17)	0.2768 (14)	0.064 (4)	
H21	0.595654	0.829418	0.272967	0.077*	
C22	0.4762 (9)	0.3628 (13)	0.0751 (9)	0.046 (2)	
H22	0.526849	0.295544	0.023314	0.055*	

### Ferrocenecarboxaldehyde Atomic displacement parameters (Å^2^)

**Table:**

	*U*^11^	*U*^22^	*U*^33^	*U*^12^	*U*^13^	*U*^23^
Fe1	0.0255 (5)	0.0258 (8)	0.0489 (6)	−0.0017 (5)	0.0031 (4)	−0.0006 (5)
Fe2	0.0253 (6)	0.0246 (7)	0.0565 (7)	−0.0025 (5)	0.0006 (5)	0.0015 (5)
O11	0.089 (7)	0.120 (10)	0.061 (6)	−0.062 (6)	0.015 (4)	−0.005 (5)
O22	0.049 (4)	0.044 (4)	0.068 (4)	−0.002 (3)	−0.006 (3)	0.004 (3)
C1	0.031 (5)	0.030 (5)	0.062 (6)	−0.011 (4)	0.007 (4)	−0.002 (4)
C2	0.035 (6)	0.030 (6)	0.071 (7)	−0.007 (4)	−0.003 (4)	0.012 (5)
C3	0.046 (7)	0.039 (6)	0.092 (9)	−0.011 (5)	0.011 (6)	−0.030 (6)
C4	0.060 (7)	0.083 (9)	0.051 (6)	−0.036 (6)	0.001 (5)	−0.015 (6)
C5	0.030 (5)	0.039 (6)	0.079 (7)	−0.010 (4)	−0.009 (4)	0.007 (5)
C6	0.039 (5)	0.045 (7)	0.063 (6)	−0.010 (5)	0.011 (4)	−0.015 (6)
C7	0.034 (5)	0.040 (6)	0.051 (5)	−0.015 (4)	−0.008 (3)	0.001 (4)
C8	0.028 (4)	0.022 (5)	0.065 (6)	−0.007 (3)	0.011 (3)	0.002 (4)
C9	0.030 (5)	0.035 (6)	0.068 (6)	−0.009 (4)	0.001 (4)	0.012 (5)
C10	0.039 (6)	0.014 (6)	0.100 (9)	0.001 (4)	0.013 (5)	0.001 (5)
C11	0.043 (6)	0.052 (7)	0.084 (8)	−0.021 (5)	0.015 (5)	−0.011 (6)
C12	0.026 (4)	0.036 (6)	0.054 (5)	−0.005 (4)	0.000 (3)	−0.002 (4)
C13	0.035 (5)	0.021 (5)	0.083 (8)	−0.005 (4)	0.016 (5)	0.003 (5)
C14	0.036 (5)	0.047 (6)	0.071 (7)	−0.010 (5)	−0.003 (4)	0.014 (5)
C15	0.039 (5)	0.055 (7)	0.054 (6)	−0.013 (5)	0.010 (4)	−0.002 (5)
C16	0.041 (5)	0.042 (6)	0.054 (5)	−0.009 (4)	0.010 (4)	−0.002 (5)
C17	0.048 (6)	0.038 (7)	0.072 (7)	−0.021 (5)	−0.009 (5)	0.022 (6)
C18	0.031 (4)	0.042 (6)	0.065 (6)	−0.004 (4)	0.007 (3)	0.017 (5)
C19	0.037 (5)	0.049 (7)	0.065 (6)	−0.007 (4)	−0.011 (4)	0.006 (5)
C20	0.043 (6)	0.039 (7)	0.089 (8)	−0.025 (5)	−0.001 (5)	−0.010 (6)
C21	0.035 (6)	0.028 (7)	0.128 (11)	−0.007 (5)	0.002 (6)	0.013 (7)
C22	0.049 (6)	0.028 (5)	0.062 (6)	−0.012 (4)	0.011 (4)	−0.009 (4)

### Ferrocenecarboxaldehyde Geometric parameters (Å, º)

**Table:**

Fe1—C1	2.021 (9)	C6—H6	0.9500
Fe1—C2	2.053 (10)	C6—C7	1.391 (14)
Fe1—C3	2.050 (10)	C6—C10	1.424 (16)
Fe1—C4	2.056 (10)	C7—H7	0.9500
Fe1—C5	2.016 (9)	C7—C8	1.394 (12)
Fe1—C6	2.045 (11)	C8—H8	0.9500
Fe1—C7	2.047 (8)	C8—C9	1.411 (13)
Fe1—C8	2.046 (8)	C9—H9	0.9500
Fe1—C9	2.034 (10)	C9—C10	1.413 (16)
Fe1—C10	2.029 (11)	C10—H10	0.9500
Fe2—C12	2.038 (9)	C11—H11	0.9500
Fe2—C13	2.021 (10)	C12—C13	1.437 (14)
Fe2—C14	2.056 (11)	C12—C16	1.423 (14)
Fe2—C15	2.045 (9)	C12—C22	1.442 (14)
Fe2—C16	2.043 (9)	C13—H13	0.9500
Fe2—C17	2.031 (10)	C13—C14	1.396 (16)
Fe2—C18	2.041 (8)	C14—H14	0.9500
Fe2—C19	2.040 (9)	C14—C15	1.416 (15)
Fe2—C20	2.035 (11)	C15—H15	0.9500
Fe2—C21	2.028 (13)	C15—C16	1.417 (14)
O11—C11	1.137 (14)	C16—H16	0.9500
O22—C22	1.229 (12)	C17—H17	0.9500
C1—C2	1.440 (14)	C17—C18	1.417 (15)
C1—C5	1.403 (15)	C17—C21	1.393 (19)
C1—C11	1.451 (16)	C18—H18	0.9500
C2—H2	0.9500	C18—C19	1.399 (14)
C2—C3	1.384 (16)	C19—H19	0.9500
C3—H3	0.9500	C19—C20	1.399 (16)
C3—C4	1.392 (19)	C20—H20	0.9500
C4—H4	0.9500	C20—C21	1.417 (18)
C4—C5	1.408 (17)	C21—H21	0.9500
C5—H5	0.9500	C22—H22	0.9500
			
C1—Fe1—C2	41.4 (4)	Fe1—C4—H4	127.4
C1—Fe1—C3	67.7 (4)	C3—C4—Fe1	69.9 (6)
C1—Fe1—C4	67.7 (4)	C3—C4—H4	126.0
C1—Fe1—C6	109.0 (4)	C3—C4—C5	108.0 (10)
C1—Fe1—C7	128.8 (4)	C5—C4—Fe1	68.3 (6)
C1—Fe1—C8	166.0 (4)	C5—C4—H4	126.0
C1—Fe1—C9	152.3 (4)	Fe1—C5—H5	124.4
C1—Fe1—C10	118.5 (4)	C1—C5—Fe1	69.8 (5)
C2—Fe1—C4	67.0 (5)	C1—C5—C4	107.9 (10)
C3—Fe1—C2	39.4 (5)	C1—C5—H5	126.1
C3—Fe1—C4	39.6 (5)	C4—C5—Fe1	71.3 (5)
C5—Fe1—C1	40.7 (4)	C4—C5—H5	126.1
C5—Fe1—C2	68.6 (4)	Fe1—C6—H6	126.5
C5—Fe1—C3	67.7 (4)	C7—C6—Fe1	70.2 (6)
C5—Fe1—C4	40.4 (5)	C7—C6—H6	125.9
C5—Fe1—C6	129.6 (4)	C7—C6—C10	108.1 (9)
C5—Fe1—C7	166.9 (4)	C10—C6—Fe1	68.9 (6)
C5—Fe1—C8	152.0 (4)	C10—C6—H6	125.9
C5—Fe1—C9	118.7 (4)	Fe1—C7—H7	125.9
C5—Fe1—C10	108.5 (4)	C6—C7—Fe1	70.1 (5)
C6—Fe1—C2	118.8 (4)	C6—C7—H7	125.6
C6—Fe1—C3	151.3 (5)	C6—C7—C8	108.8 (9)
C6—Fe1—C4	167.9 (5)	C8—C7—Fe1	70.0 (4)
C6—Fe1—C7	39.7 (4)	C8—C7—H7	125.6
C6—Fe1—C8	67.2 (4)	Fe1—C8—H8	126.1
C7—Fe1—C2	108.3 (4)	C7—C8—Fe1	70.1 (5)
C7—Fe1—C3	118.6 (5)	C7—C8—H8	126.0
C7—Fe1—C4	151.3 (5)	C7—C8—C9	108.0 (9)
C8—Fe1—C2	127.3 (4)	C9—C8—Fe1	69.3 (5)
C8—Fe1—C3	108.6 (4)	C9—C8—H8	126.0
C8—Fe1—C4	118.8 (4)	Fe1—C9—H9	125.9
C8—Fe1—C7	39.8 (4)	C8—C9—Fe1	70.2 (5)
C9—Fe1—C2	165.0 (4)	C8—C9—H9	126.0
C9—Fe1—C3	128.6 (5)	C8—C9—C10	108.0 (9)
C9—Fe1—C4	109.1 (5)	C10—C9—Fe1	69.5 (6)
C9—Fe1—C6	68.0 (5)	C10—C9—H9	126.0
C9—Fe1—C7	67.6 (4)	Fe1—C10—H10	125.1
C9—Fe1—C8	40.5 (4)	C6—C10—Fe1	70.2 (6)
C10—Fe1—C2	152.8 (4)	C6—C10—H10	126.5
C10—Fe1—C3	166.6 (6)	C9—C10—Fe1	69.8 (6)
C10—Fe1—C4	129.3 (5)	C9—C10—C6	107.0 (9)
C10—Fe1—C6	40.9 (5)	C9—C10—H10	126.5
C10—Fe1—C7	68.0 (5)	O11—C11—C1	130.2 (13)
C10—Fe1—C8	68.2 (4)	O11—C11—H11	114.9
C10—Fe1—C9	40.7 (5)	C1—C11—H11	114.9
C12—Fe2—C14	68.6 (4)	C13—C12—Fe2	68.7 (5)
C12—Fe2—C15	68.4 (4)	C13—C12—C22	124.6 (9)
C12—Fe2—C16	40.8 (4)	C16—C12—Fe2	69.8 (5)
C12—Fe2—C18	122.9 (4)	C16—C12—C13	106.7 (9)
C12—Fe2—C19	158.2 (4)	C16—C12—C22	128.2 (10)
C13—Fe2—C12	41.5 (4)	C22—C12—Fe2	120.6 (7)
C13—Fe2—C14	40.0 (5)	Fe2—C13—H13	124.9
C13—Fe2—C15	67.9 (4)	C12—C13—Fe2	69.9 (6)
C13—Fe2—C16	68.7 (4)	C12—C13—H13	125.5
C13—Fe2—C17	125.8 (5)	C14—C13—Fe2	71.3 (7)
C13—Fe2—C18	108.5 (5)	C14—C13—C12	109.0 (9)
C13—Fe2—C19	122.0 (4)	C14—C13—H13	125.5
C13—Fe2—C20	156.0 (5)	Fe2—C14—H14	127.4
C13—Fe2—C21	162.0 (5)	C13—C14—Fe2	68.6 (6)
C15—Fe2—C14	40.4 (4)	C13—C14—H14	126.1
C16—Fe2—C14	68.3 (4)	C13—C14—C15	107.7 (9)
C16—Fe2—C15	40.6 (4)	C15—C14—Fe2	69.4 (6)
C17—Fe2—C12	108.6 (4)	C15—C14—H14	126.1
C17—Fe2—C14	161.5 (5)	Fe2—C15—H15	126.1
C17—Fe2—C15	157.2 (5)	C14—C15—Fe2	70.2 (5)
C17—Fe2—C16	122.4 (4)	C14—C15—H15	125.7
C17—Fe2—C18	40.7 (4)	C14—C15—C16	108.7 (9)
C17—Fe2—C19	67.5 (4)	C16—C15—Fe2	69.6 (5)
C17—Fe2—C20	68.2 (5)	C16—C15—H15	125.7
C18—Fe2—C14	124.2 (5)	Fe2—C16—H16	126.3
C18—Fe2—C15	160.0 (4)	C12—C16—Fe2	69.4 (5)
C18—Fe2—C16	158.4 (4)	C12—C16—H16	126.1
C19—Fe2—C14	107.8 (4)	C15—C16—Fe2	69.8 (5)
C19—Fe2—C15	123.7 (4)	C15—C16—C12	107.9 (9)
C19—Fe2—C16	159.7 (4)	C15—C16—H16	126.1
C19—Fe2—C18	40.1 (4)	Fe2—C17—H17	125.9
C20—Fe2—C12	160.5 (4)	C18—C17—Fe2	70.0 (6)
C20—Fe2—C14	120.8 (5)	C18—C17—H17	125.9
C20—Fe2—C15	106.7 (5)	C21—C17—Fe2	69.8 (7)
C20—Fe2—C16	123.3 (4)	C21—C17—H17	125.9
C20—Fe2—C18	68.2 (5)	C21—C17—C18	108.3 (10)
C20—Fe2—C19	40.2 (5)	Fe2—C18—H18	125.9
C21—Fe2—C12	124.3 (5)	C17—C18—Fe2	69.3 (5)
C21—Fe2—C14	156.6 (6)	C17—C18—H18	126.5
C21—Fe2—C15	121.5 (5)	C19—C18—Fe2	69.9 (5)
C21—Fe2—C16	107.6 (5)	C19—C18—C17	107.0 (10)
C21—Fe2—C17	40.2 (6)	C19—C18—H18	126.5
C21—Fe2—C18	68.1 (5)	Fe2—C19—H19	126.6
C21—Fe2—C19	67.6 (5)	C18—C19—Fe2	70.0 (5)
C21—Fe2—C20	40.8 (5)	C18—C19—H19	125.3
C2—C1—Fe1	70.5 (5)	C18—C19—C20	109.4 (10)
C2—C1—C11	127.8 (10)	C20—C19—Fe2	69.7 (5)
C5—C1—Fe1	69.5 (5)	C20—C19—H19	125.3
C5—C1—C2	107.5 (9)	Fe2—C20—H20	125.6
C5—C1—C11	124.2 (11)	C19—C20—Fe2	70.1 (7)
C11—C1—Fe1	119.0 (7)	C19—C20—H20	126.5
Fe1—C2—H2	126.8	C19—C20—C21	106.9 (12)
C1—C2—Fe1	68.1 (6)	C21—C20—Fe2	69.3 (7)
C1—C2—H2	126.5	C21—C20—H20	126.5
C3—C2—Fe1	70.2 (6)	Fe2—C21—H21	125.9
C3—C2—C1	106.9 (9)	C17—C21—Fe2	70.0 (7)
C3—C2—H2	126.5	C17—C21—C20	108.4 (11)
Fe1—C3—H3	125.6	C17—C21—H21	125.8
C2—C3—Fe1	70.4 (6)	C20—C21—Fe2	69.9 (7)
C2—C3—H3	125.2	C20—C21—H21	125.8
C2—C3—C4	109.6 (10)	O22—C22—C12	125.6 (9)
C4—C3—Fe1	70.4 (7)	O22—C22—H22	117.2
C4—C3—H3	125.2	C12—C22—H22	117.2
			
Fe1—C1—C2—C3	59.7 (7)	C7—C6—C10—C9	1.0 (12)
Fe1—C1—C5—C4	−61.5 (7)	C7—C8—C9—Fe1	59.7 (6)
Fe1—C1—C11—O11	102.6 (13)	C7—C8—C9—C10	0.3 (11)
Fe1—C2—C3—C4	59.7 (8)	C8—C9—C10—Fe1	59.9 (7)
Fe1—C3—C4—C5	57.8 (8)	C8—C9—C10—C6	−0.8 (12)
Fe1—C4—C5—C1	60.6 (7)	C10—C6—C7—Fe1	58.7 (8)
Fe1—C6—C7—C8	−59.5 (7)	C10—C6—C7—C8	−0.8 (12)
Fe1—C6—C10—C9	60.4 (7)	C11—C1—C2—Fe1	112.2 (10)
Fe1—C7—C8—C9	−59.2 (6)	C11—C1—C2—C3	171.9 (10)
Fe1—C8—C9—C10	−59.4 (7)	C11—C1—C5—Fe1	−111.9 (9)
Fe1—C9—C10—C6	−60.7 (7)	C11—C1—C5—C4	−173.4 (9)
Fe2—C12—C13—C14	60.8 (7)	C12—C13—C14—Fe2	−60.0 (7)
Fe2—C12—C16—C15	−59.4 (7)	C12—C13—C14—C15	−1.4 (11)
Fe2—C12—C22—O22	−93.4 (11)	C13—C12—C16—Fe2	59.0 (6)
Fe2—C13—C14—C15	58.6 (7)	C13—C12—C16—C15	−0.4 (11)
Fe2—C14—C15—C16	59.2 (7)	C13—C12—C22—O22	−177.4 (9)
Fe2—C15—C16—C12	59.2 (7)	C13—C14—C15—Fe2	−58.1 (7)
Fe2—C17—C18—C19	−60.1 (7)	C13—C14—C15—C16	1.1 (12)
Fe2—C17—C21—C20	59.6 (8)	C14—C15—C16—Fe2	−59.6 (7)
Fe2—C18—C19—C20	−58.7 (7)	C14—C15—C16—C12	−0.4 (11)
Fe2—C19—C20—C21	−59.8 (7)	C16—C12—C13—Fe2	−59.7 (7)
Fe2—C20—C21—C17	−59.7 (8)	C16—C12—C13—C14	1.1 (11)
C1—C2—C3—Fe1	−58.4 (7)	C16—C12—C22—O22	−6.0 (16)
C1—C2—C3—C4	1.3 (12)	C17—C18—C19—Fe2	59.6 (7)
C2—C1—C5—Fe1	60.6 (6)	C17—C18—C19—C20	1.0 (12)
C2—C1—C5—C4	−1.0 (11)	C18—C17—C21—Fe2	−59.6 (8)
C2—C1—C11—O11	15.6 (18)	C18—C17—C21—C20	−0.1 (12)
C2—C3—C4—Fe1	−59.7 (8)	C18—C19—C20—Fe2	58.8 (7)
C2—C3—C4—C5	−1.9 (13)	C18—C19—C20—C21	−1.0 (12)
C3—C4—C5—Fe1	−58.8 (8)	C19—C20—C21—Fe2	60.4 (8)
C3—C4—C5—C1	1.8 (12)	C19—C20—C21—C17	0.7 (12)
C5—C1—C2—Fe1	−59.9 (6)	C21—C17—C18—Fe2	59.5 (8)
C5—C1—C2—C3	−0.2 (11)	C21—C17—C18—C19	−0.5 (12)
C5—C1—C11—O11	−173.5 (10)	C22—C12—C13—Fe2	113.2 (9)
C6—C7—C8—Fe1	59.5 (7)	C22—C12—C13—C14	174.1 (9)
C6—C7—C8—C9	0.3 (11)	C22—C12—C16—Fe2	−113.6 (10)
C7—C6—C10—Fe1	−59.5 (8)	C22—C12—C16—C15	−173.0 (9)

### Ferrocenecarboxaldehyde Hydrogen-bond geometry (Å, º)

*Cg*1–*Cg*4 are the centroids of the (C1–C5), (C6–C10), (C12–C16) and (C17–C21) rings, respectively.

**Table:**

*D*—H···*A*	*D*—H	H···*A*	*D*···*A*	*D*—H···*A*
C3—H3···O22^i^	0.95	2.60	3.520 (13)	165
C6—H6···O11^ii^	0.95	2.45	3.255 (13)	142
C7—H7···*Cg*3	0.95	2.91	3.625 (12)	133
C11—H11···*Cg*4^iii^	0.95	2.97	3.907 (12)	170
C16—H16···*Cg*2^iv^	0.95	3.03	3.935 (13)	159
C18—H18···*Cg*1^v^	0.95	2.80	3.631 (7)	146

Symmetry codes: (i) −*x*+1, *y*+1/2, −*z*+1; (ii) −*x*+2, *y*−1/2, −*z*+1; (iii) *x*−1, *y*−1, *z*; (iv) *x*, *y*+1, *z*; (v) *x*+1, *y*, *z*.
